# Hemodynamics and vasopressor support during targeted temperature management after cardiac arrest with non-shockable rhythm: A post hoc analysis of a randomized controlled trial

**DOI:** 10.1016/j.resplu.2022.100271

**Published:** 2022-07-12

**Authors:** Matthieu Petit, Jean-Baptiste Lascarrou, Gwenhael Colin, Hamid Merdji, Alain Cariou, Guillaume Geri, HYPERION investigators

**Affiliations:** a Medical Intensive Care Unit, Ambroise Paré Hospital, APHP, Boulogne-Billancourt, France; b Paris-Saclay University, UVSQ, Inserm, CESP, 94807 Villejuif, France; c Médecine Intensive Réanimation, University Hospital Center, Nantes, France; d Paris Cardiovascular Research Center, INSERM U970, Paris, France; e AfterROSC Network, France; f Medical-Surgical Intensive Care Unit, District Hospital Center, La Roche-sur-Yon, France; g Université de Strasbourg (UNISTRA), Faculté de Médecine, Hôpitaux universitaires de Strasbourg, Nouvel Hôpital Civil, Service de Médecine Intensive Réanimation, Strasbourg, France; h UMR 1260, Regenerative Nano Medecine, INSERM, Fédération de Médecine Translationnelle de Strasbourg (FMTS), Université de Strasbourg, Strasbourg, France; i Medical Intensive Care Unit, Cochin University Hospital Center, Paris, France; j Medical and Surgical Intensive Care Unit, Ambroise Paré Clinic, Neuilly-sur-Seine, France

**Keywords:** Cardiac arrest, Targeted temperature management, Post resuscitation shock, Circulatory failure

## Abstract

**Background:**

Patients admitted after cardiac arrest with non-shockable rhythm frequently experience hemodynamic instability. This study assessed the hemodynamic consequences of TTM in this sub population.

**Methods:**

This is a post hoc analysis of the HYPERION trial (NCT01994772), that randomized patients to either hypothermia or normothermia after non-shockable rhythm related cardiac arrest. Patients with no, moderate or severe circulatory failure were identified with cardiovascular Sequential Organ Failure Assessment at randomization. Primary outcome was the number of patients at day 7 with resolution of shock, accounting for the risk of death (competing risk analysis). Secondary endpoint included neurological outcome and death at day-90.

**Results:**

584 patients were included in the analysis: 195 (34%), 46 (8%) and 340 (59%) had no, moderate and severe circulatory failure, respectively. Resolution of circulatory failure at day 7 was more frequently observed in the normothermia group than in the TTM group (60% [95 %CI 54–66] versus 53% [95 %CI 46–60], Gray-test: p = 0.016). The severity of circulatory failure at randomization was associated with its less frequent resolution at day 7 accounting for the risk of death (76 % [62–86] versus 54% [49–59] for patients with moderate versus severe circulatory failure, Gray test, p < 0.001, respectively). At day 90, the proportion of patients with Cerebral Performance Category score of 1 or 2 was lower in patients presenting severe circulatory failure (p = 0.038).

**Conclusion:**

Circulatory failure is frequent after CA with non-shockable rhythm. Its severity at admission and TTM were associated with delayed resolution of circulatory failure.

## Introduction

In consequence of whole body ischemia–reperfusion syndrome subsequent to cardiac arrest (CA), successfully resuscitated patients often develop severe post-resuscitation circulatory failure,1., 2. associating vasoplegia, myocardial dysfunction and systemic ischemic injury.3., 4., 5. This circulatory failure, included in the well-known “post-resuscitation disease”, may be associated with different other organ failure and drastically impacts outcome.6., 7. Targeted temperature management (TTM) is so far recommended by international guidelines^8^ to prevent or limit hypoxic-ischemic brain damage in CA patients remaining comatose after restoration of spontaneous circulation resuscitation (ROSC).^9^ Circulatory failure may be potentially worsened by induced hypotermia, which is potentially a stress to the cardiovascular system with significant hemodynamic impact involving decreased cardiac index, lower heart rate and increased systemic vascular resistance.^3^ While there is a lot of experimental and animal data10., 11., 12., 13. on the impact of temperature management and hemodynamics,12., 13., 14. very few data have been published so far to describe the potential longitudinal impact of TTM on early hemodynamics of resuscitated CA patients. The HYPERION trial^15^ recently provided data in favor of hypothermia in CA patients with initial non-shockable rhythm. Impact of such a strategy on hemodynamic parameters in patients presenting post-resuscitation circulatory failure has not been fully studied in this situation.

In the present study, using the Hyperion trial data, we aimed to investigate cardiovascular consequences of hypothermia in CA patients with non-shockable rhythm and its association with outcome.

## Methods

### Design

This was a post-hoc analysis of the Hyperion trial (NCT01994772), a multicentric French randomized controlled trial investigating TTM at 33°c versus normothermia during the first 24 hours in comatose (score ≤ 8 on the Glasgow Coma Scale) CA patients with non-shockable rhythm survivors.^15^

### Patients

In this post hoc analysis, all the patients included in the Hyperion trial were included. Severity of circulatory failure at admission was defined with the cardiovascular Sequential Organ Failure Assessment (cSOFA),^16^ and patient were divided in 3 groups: no (cSOFA equal to 0 or 1, no vasopressor support), moderate (cSOFA equal to 2 or 3, inotrope support or low dose vasopressor support < 0.5 µg/kg/min) and severe circulatory failure (cSOFA equal to 4, high dose of vasopressor support > 0.5 µg/kg/min).

According to the French law, because the strategies used in both groups were considered to be components of standard care, informed consent for trial participation was not required. However, French data-protection authorities require that patients be given the opportunity to decline that their data to be used. Therefore, since the patients had coma, it was required that the closest available relatives received specific information about trial enrollment. Patients with no available relative were included in the trial, informed as soon as they regained competence, and were asked whether they wanted to remain in the trial; if the answer was negative, they were excluded from the analysis. Approval of the ethical comitee of the French Society of Intensive Care Medicine was obtained (CE-SRLF-11-335).

### Intervention

Patients were randomized to intervention groups of moderate therapeutic hypothermia (33 °C) or targeted normothermia (37 °C). In the hypothermia group, hypothermia at 33 °C was induced and maintained for 24 hours; an then slow rewarming with a maximum speed of 0.5°/hour was performed to 36.5 to 37.5 °C, and maintained for 24 hours. In the normothermia group, body temperature was maintained at 36.5 to 37.5 °C for 48 hours. Hemodynamic evaluations were conducted to allow blood volume optimization. Hypovolemia was managed with crystalloid or colloid infusion, according to standard practice in each participating intensive care unit (ICU). The introduction of vasoactive drug treatment was at the discretion of the physicians, who followed international guidelines and local protocols. A mean arterial pressure of 65 mmHg and, if measured, central venous oxygen saturation (ScvO2) ≥ 70% were considered reasonable targets.

### Endpoints

The primary endpoint of the present study was the number of patient with circulatory failure resolution at day 7. Resolution of shock was defined as the definite weaning of both vasopressors and inotropes for at least 24 hours, identified by a cSOFA equal to 0.

Secondary endpoints included the timing of circulator failure resolution, the association of circulatory failure with the Cerebral Performance Category (CPC) score on day 90 as well as day-90 mortality.

## Statistical analysis

Continuous variables were expressed as median (1st–3rd quartiles) while categorical variables were expressed as frequencies (percentage). Baseline characteristics were compared across cSOFA categories using ordered Cochrane-Armitage test and Jonckheere’s test for categorical and continuous variables, respectively.

The primary outcome was assessed using a competitive risk analysis and compared with a Gray Test. Indeed, the event of interest was the resolution of shock during the first 7 days. This outcome is impacted by the time of onset of the event of interest. Patients who have not experienced the event at the end of follow-up (day 7) were censored. To determine the risk of an event occurring at a certain time-point, a fundamental assumption is the outcome is not associated with an altered chance of the event occuring at any given moment. Death (from any causes) before resolution of shock is a competing event. For that puprpose, outcome was assessed using a competing risk model (Gray test, and cumulative incidence curves).

All statistical tests were two-sided, with p ≤ 0.05 considered significant. Statistical analysis was computed with R software (Version 3.6.3; R Foundation for Statistical Computing, Vienna, Austria) and the statistical package Crmpsk for the competing risk analysis.

## Results

### Patients

**Fig. 1:**
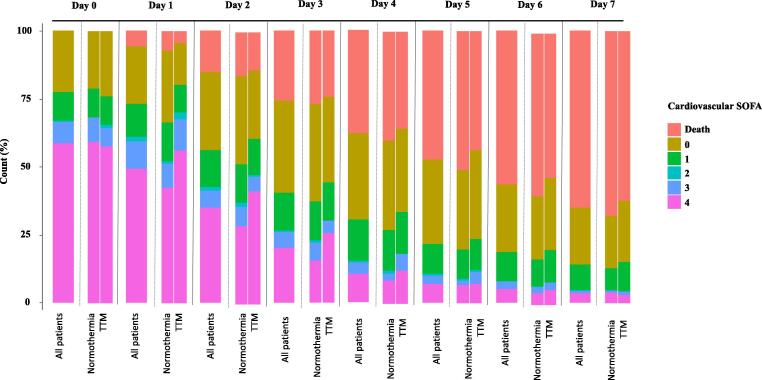
**Evolution of cardiovascular SOFA over time according to group of randomization.** SOFA Sequential organ failure asssessment, TTM Targeted temperature management (hypothermia).

All of the 581 patients included in the Hyperion trial were included in this post-hoc analysis: 284 allocated to TTM and 297 to normothermia. At inclusion, 195 (34%), 46 (8%) and 340 (59%) patients had no, moderate and severe circulatory failure, respectively. Baseline characteristics were not different between the 3 groups (Table1), except for a statistically longer no-flow time in patients who did not have circulatory failure (p = 0.045), a lower temperature at enrollment (p = 0.035) and a more frequent use of epinephrine (p = 0.012) in patients with severe circulatory failure. Proportions of patients receiving inotrope or vasopressor for the first 7 days after CA according to group of randomization are given in Fig. 1**.** Baseline characteristics and outcome of these patients are described in ESM Table 1. Hemodynamics variables and temperature during the first 68 hours of management are given in ESM Fig. 1. Twenty patients developed circulatory failure after the period of intervention (ESM Table 2).

**Table 1:** Baseline characteristics of the patients enrolled in the study according to the shock status at admission.

	Overall	No shock	Moderate shock	Severe shock	P_trend_
n	581	195	46	340	
Age - years	67.00 [57.00, 76.00]	65.00 [55.00, 74.00]	65.00 [55.50, 77.75]	67.00 [58.00, 76.00]	0.977
Sexe = male (%)	373 (64.2)	131 (67.2)	30 (65.2)	212 (62.4)	0.129
Charlson score	1.00 [0.00, 3.00]	1.00 [0.00, 3.00]	2.00 [1.00, 3.75]	1.00 [0.00, 3.00]	0.935
Chronic heart failure	66 (11.4)	20 (10.3)	6 (13.0)	40 (11.8)	0.618
Chronic respiratory disease	204 (47.0)	59 (44.4)	18 (42.9)	127 (49.0)	0.351
Location of cardiac arrest					0.378
Home	295 (50.8)	106 (54.4)	20 (43.5)	169 (49.7)	
Hospital	159 (27.4)	45 (23.1)	13 (28.3)	101 (29.7)	
Public place	127 (21.9)	44 (22.6)	13 (28.3)	70 (20.6)	
Bystander-witnessed cardiac arrest	204 (47.0)	59 (44.4)	18 (42.9)	127 (49.0)	0.579
Bystander-performed CPR	407 (74.4)	128 (70.3)	31 (72.1)	248 (77.0)	0.239
Cause of cardiac arrest					0.141
Asphyxia	290 (49.9)	103 (52.8)	20 (43.5)	167 (49.1)	
Other medical cause	82 (14.1)	23 (11.8)	6 (13.0)	53 (15.6)	
Cardiac cause	153 (26.3)	49 (25.1)	17 (37.0)	87 (25.6)	
Pulmonary embolism	21 (3.6)	4 (2.1)	1 (2.2)	16 (4.7)	
Neurological cause	13 (2.2)	5 (2.6)	0 (0.0)	8 (2.4)	
Drowning	6 (1.0)	1 (0.5)	1 (2.2)	4 (1.2)	
Hanging	13 (2.2)	9 (4.6)	0 (0.0)	4 (1.2)	
Trauma	3 (0.5)	1 (0.5)	1 (2.2)	1 (0.3)	
No_flow - min	2.00 [0.00, 5.00]	3.00 [0.00, 7.00]	1.00 [0.00, 5.00]	1.00 [0.00, 5.00]	0.033
Low_flow - min	16.00 [10.00, 25.00]	16.00 [10.00, 25.00]	19.50 [9.25, 25.75]	15.50 [10.00, 26.25]	0.806
Use of epinephrin	535 (92.1)	173 (88.7)	42 (91.3)	320 (94.1)	0.012
Coronary angiogrpahy	148 (25.5)	46 (23.6)	11 (23.9)	91 (26.8)	0.407
Temperature at enrollment - °C	35.5 [34.5, 36.4]	35.60 [34.8, 36.5]	35.55 [34.4, 36.5]	35.40 [34.3, 36.4]	0.035
Hypothermia	284 (48.9)	101 (51.8)	19 (41.3)	164 (48.2)	0.480

Categorical variables are expressed as number (%) and continous variables as median (interquartile ranges).

SOFA, Sequential organ failure assessment.

### Primary outcome

**Fig. 2:**
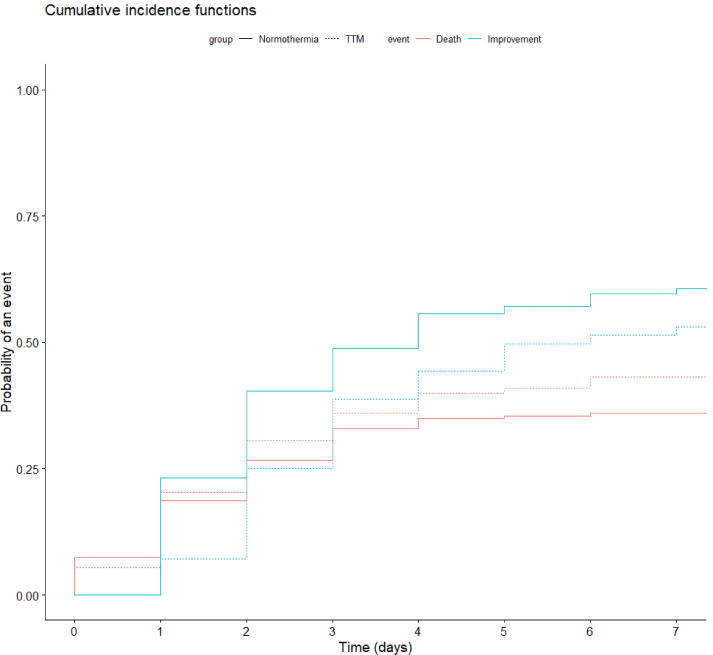
**Cumulative incidences of death and haemodynamic improvement over time according to the group of randomization.** These cumulative incidence functions were obtained after competitive risk analysis and were statistically different concerning the timing of resolution of circulatory failure (gray test, p = 0,016) while the probabilty of death. TTM Targeted temperature management (hypothermia).

Among patients presenting circulatory failure at admission, resolution of shock at day-7 was more frequently observed in the normothermia than in the TTM group (60% [95 %CI 54–66] versus 53% [95 %CI 46–60], Gray-test: p = 0.016) (Fig. 2). Mortality at day 7 was similar in TTM group and normothermia group (36% [95 %CI 30–42] versus 43% [95 %CI 36–50], Gray-test: p = 0.18). Among survivors at day 7, median duration of circulatory failure was 2 [1;3] days in normothermia group versus 3 [2;4] days in TTM group (p < 0.001). The severity of circulatory failure at randomization was associated with a lower resolution of shock at day 7 accounting for the risk of death (76 % [95 %CI 62–86] for patients with moderate shock, versus 54% [95 %CI 49–59] for patients with severe shock, gray test, p < 0.001).

### Secondary outcome

TTM interruption was not more frequent in patients presenting the most severe circulatory failure and was observed in 22 (13.4%), 4 (21.1%) and 10 (9.9%) patients with severe, moderate and no circulatory failure at admission, respectively (p = 0.370).

At day-90, survival was similar in the 3 groups (p = 0.49, Table 2). Proportion of patients with a CPC score of 1 or 2 at day 90 was lower in patients presenting severe circulatory failure at randomization (p = 0.038).

**Table 2:** Outcome of the patients enrolled in the study according to the shock status at admission.

	No shock	Moderate shock	Severe shock	P_trend_
n	195	46	340	
Brain death	33 (20.1)	7 (20.0)	53 (18.7)	0.356
CPC score of 1 or 2 on day 90	12 (6.2)	8 (17.4)	26 (7.6)	0.038
CPC score distribution on day 90				0.479
1	7 (3.6)	5 (10.9)	15 (4.4)	
2	5 (2.6)	3 (6.5)	11 (3.2)	
3	20 (10.3)	3 (6.5)	30 (8.8)	
4	0 (0.0)	0 (0.0)	1 (0.3)	
5	163 (83.6)	35 (76.1)	283 (83.2)	
Death by day 90	160 (82.1)	35 (76.1)	283 (83.2)	0.332

Categorical variables are expressed as number (%) and continous variables as median (interquartile ranges).

CPC, Cerebral performance category.

## Discussion

In this post hoc analysis of the Hyperion trial involving 581 comatose patients after CA with non-shockable rhythm admitted to the ICU, we compared outcomes over severity of post-resuscitation circulatory failure within the first 7 days after ICU admission. To our knowledge, only few small studies have investigated hemodynamic during TTM in this subgroup of patients. We found that 1) while heart rate was significantly lower in patient with TTM, MAP was similar between the two groups of randomization 2) 66 % of patients evidenced post-resuscitation circulatory failure at randomization, and the severity of shock at admission and the intervention were associated with a delay in the timing of hemodynamic improvement 3) severity of shock at admission was associated with a worse functional outcome at day 90 evaluated with CPC score.

Post-CA syndrome is frequent in CA survivors resuscitated from initial non-shockable rhythm.1., 2., 17. Its pathogenesis is complex, involving at different levels ischemia reperfusion syndrome^1^ worsened by the common hypoxic etiology^15^ as well as infectious phenomena and myocardial dysfunction. Consequent systemic inflammation induces vasodilatation and lower MAP 1,^17^ motivating vasopressor infusion to avoid subsequent cerebral aggression,5., 17. which may drastically grave the functional outcome of these patients^7^. Precedent studies investigated the hemodynamic implication of TTM and found that several factors were associated with higher need of vasopressor,3., 6., 19., 20. as time to return to spontaneous circulation, age, or percutaneous coronary intervention. While the circulatory failure after ROSC is now well identified as a factor of poor prognosis,^18^ the impact of TTM in these patients, potentially worsening the shock, is still unknown. Moreover, these moribund/instable patients are very frequently excluded from these studies, precluding us to drive conclusions.9., 21. In non-shockable rhythm CA survivors admitted to the ICU, we found that the severity of shock at admission was associated with a worse functional outcome at day-90. Furthermore, TTM delay the timing of shock resolution at day-7 although the rate of TTM interruption was similar in the three subgroups. Grand et al.,^5^ found similar findings in patient with prolonged TTM in their post-hoc analysis of the TT48 trial. Similar results have been described in a retrospective registry-based study of 412 CA patients.^6^

In these situation, TTM may worsen the hemodynamic status, and consequently, could alter oxygen delivery to the brain. Indeed, hypothermia has negative hemodynamic effects by slowering metabolism and directly depressing myocardial function.^22^ Patients can developped hypovolemia in consequence of an augmentation of urine output (also called «cold diuresis»).^23^ Afterload is also raised through the release of catecholamins, leading to an increase of arterial resistance.^22^ As a result of all these mechanisms, cardiac output is decreased.^22^ Finally, it has been reported that, in patients with pre-existent coronary artery disease, coronary vasconstriction may occur during hypothermia,^24^ because of an endothelial dysfunction, and worsen oxygen delivery to heart. Then, whether to apply or not such a strategy in these patients remains a matter of debate, especially since the results of the TTM2 trial^9^ in which the authors found discordant results in the subgroup of patients suffering cardiovascular failure at admission.

One strength of our study is the prospective and consecutive inclusion of patients and collection of data, in a multicentric randomized trial, increasing the validity of our result. However, we acknowledge that our study has several limitations. First, it was a non-prespecified post hoc analysis, and the study may have lack of power to identify association between variables and outcome. Second, some patients died during the period of analysis and hemodynamic data collection introducing a survival bias. However, the primary cause of death was withdrawal of care related to anoxic brain damage (64% of death), and we tried to limit this bias performing a competitive risk analysis, and expect the bias to be minimal. Third, the haemodynamic impact of TTM could have been more accurately evaluated using vasopressors and inotropic support doses over time as well as sedation doses. Unfortunately, these information were not collected in the HYPERION trial. While we believe this could have improved our findings, cardiovascular SOFA is a reliable tool used to evaluate one-point as well as longitudinal cardiovascular data. Finally, patients who developed circulatory failure after TTM were not analyzed as shocked patient, which can introduce a selection bias. However, we considered that this situation must not be considered as a post cardiac arrest syndrome but is due to other etiologies (sepsis, ventilator associated pneumonia, hemorrhage).

## Conclusion

Circulatory failure is frequent after CA with non-shockable rhythm survivors admitted to the ICU. Severity of circulatory failure at admission and TTM strategy were associated with worse long-term neurological outcome and a delay in the timing of resolution of circulatory failure.

## Conflict of Interest

Dr. Lascarrou reports consulting fees from BD and Zoll. None of the other authors has any conflicts of interest to disclose.

## Appendix A

List of HYPERION investigators: P. Asfar MD Medical Intensive Care Unit, University Hospital, Angers (France), F. Beloncle MD Medical Intensive Care Unit, University Hospital, Angers (France), G. Bouvier MD Medical Intensive Care Unit, University Hospital, Angers (France), N. Chudeau MD Medical Intensive Care Unit, University Hospital, Angers (France), M. Conte MD Medical Intensive Care Unit, University Hospital, Angers (France), C. Darreau MD Medical Intensive Care Unit, University Hospital, Angers (France), A. Donzeau MD Medical Intensive Care Unit, University Hospital, Angers (France), N. Hubert MD Medical Intensive Care Unit, University Hospital, Angers (France), A. Kouatchet MD Medical Intensive Care Unit, University Hospital, Angers (France), N. Lerolle MD Medical Intensive Care Unit, University Hospital, Angers (France), F. Martino MD Medical Intensive Care Unit, University Hospital, Angers (France), A. Mercat MD Medical Intensive Care Unit, University Hospital, Angers (France), S. Mortaza MD Medical Intensive Care Unit, University Hospital, Angers (France), M. Pierrot MD Medical Intensive Care Unit, University Hospital, Angers (France), T. Raveau MD Medical Intensive Care Unit, University Hospital, Angers (France), T. Reydel MD Medical Intensive Care Unit, University Hospital, Angers (France), M. Saint Martin MD Medical Intensive Care Unit, University Hospital, Angers (France), V. Souday MD Medical Intensive Care Unit, University Hospital, Angers (France), O. Baudin-Jacquemin MD Medical Intensive Care Unit, General Hospital, Angoulême (France), C. Cracco MD Medical Intensive Care Unit, General Hospital, Angoulême (France), A. Desachy MD Medical Intensive Care Unit, General Hospital, Angoulême (France), C. Lafon MD Medical Intensive Care Unit, General Hospital, Angoulême (France), S. Rouleau MD Medical Intensive Care Unit, General Hospital, Angoulême (France), D. Bougon MD Medical Intensive Care Unit, General Hospital, Annecy (France), P. Charretier MD Medical Intensive Care Unit, General Hospital, Annecy (France), R. Chouquer MD Medical Intensive Care Unit, General Hospital, Annecy (France), D. Dorez MD Medical Intensive Care Unit, General Hospital, Annecy (France), E. Escudier MD Medical Intensive Care Unit, General Hospital, Annecy (France), S. Gay MD Medical Intensive Care Unit, General Hospital, Annecy (France), S. Hautefeuille MD Medical Intensive Care Unit, General Hospital, Annecy (France), A. Hyacinthe MD Medical Intensive Care Unit, General Hospital, Annecy (France), A. Levrat MD Medical Intensive Care Unit, General Hospital, Annecy (France), M. Muller MD Medical Intensive Care Unit, General Hospital, Annecy (France), M. Sirodot MD Medical Intensive Care Unit, General Hospital, Annecy (France), D. Contou MD Medical Intensive Care Unit, General Hospital, Argenteuil (France), M. Enser MD Medical Intensive Care Unit, General Hospital, Argenteuil (France), O. Pajot MD Medical Intensive Care Unit, General Hospital, Argenteuil (France), G. Plantefeve MD Medical Intensive Care Unit, General Hospital, Argenteuil (France), M. Thirion MD Medical Intensive Care Unit, General Hospital, Argenteuil (France), J. Tirolien MD Medical Intensive Care Unit, General Hospital, Argenteuil (France), H. Mentec MD Medical Intensive Care Unit, General Hospital, Argenteuil (France), A. Ait Hssain MD Medical Intensive Care Unit, University Hospital, Clermont-Ferrand (France), C. Bachelier MD Medical Intensive Care Unit, University Hospital, Clermont-Ferrand (France), 4 P. Bertrand MD Medical Intensive Care Unit, University Hospital, Clermont-Ferrand (France), D. Brégeaud MD Medical Intensive Care Unit, University Hospital, Clermont-Ferrand (France), L. Calvet MD Medical Intensive Care Unit, University Hospital, Clermont-Ferrand (France), L . Charrier MD Medical Intensive Care Unit, University Hospital, Clermont-Ferrand (France), E. Coupez MD Medical Intensive Care Unit, University Hospital, Clermont-Ferrand (France), A. Laggoune MD Medical Intensive Care Unit, University Hospital, Clermont-Ferrand (France), G. Lardillon MD Medical Intensive Care Unit, University Hospital, Clermont-Ferrand (France), A. Lautrette MD Medical Intensive Care Unit, University Hospital, Clermont-Ferrand (France), C. Leroy MD Medical Intensive Care Unit, University Hospital, Clermont-Ferrand (France), C. Lory MD Medical Intensive Care Unit, University Hospital, Clermont-Ferrand (France), R. Malhomme MD Medical Intensive Care Unit, University Hospital, Clermont-Ferrand (France), E. Soum MD Medical Intensive Care Unit, University Hospital, Clermont-Ferrand (France), B. Souweine MD Medical Intensive Care Unit, University Hospital, Clermont-Ferrand (France), P. Andreu MD Medical Intensive Care Unit, University Hospital, Dijon (France), R. Bruyère MD Medical Intensive Care Unit, University Hospital, Dijon (France), P.E. Charles MD Medical Intensive Care Unit, University Hospital, Dijon (France), A. Dargent MD Medical Intensive Care Unit, University Hospital, Dijon (France), A. Large MD Medical Intensive Care Unit, University Hospital, Dijon (France), S. Mortreux MD Medical Intensive Care Unit, University Hospital, Dijon (France), A. Pavon MD Medical Intensive Care Unit, University Hospital, Dijon (France), S. Prin MD Medical Intensive Care Unit, University Hospital, Dijon (France), J.P. Quenot MD Medical Intensive Care Unit, University Hospital, Dijon (France), J.B. Roudaut MD Medical Intensive Care Unit, University Hospital, Dijon (France), A. Toitot MD Medical Intensive Care Unit, University Hospital, Dijon (France), M. Azais MD Medical Intensive Care Unit, District Hospital Center, La Roche sur Yon (France), K. Bachoumas MD Medical Intensive Care Unit, District Hospital Center, La Roche sur Yon (France), A. Bailly MD Medical Intensive Care Unit, District Hospital Center, La Roche sur Yon (France), L. Camous MD Medical Intensive Care Unit, District Hospital Center, La Roche sur Yon (France), G. Colin MD Medical Intensive Care Unit, District Hospital Center, La Roche sur Yon (France), L. Crosby MD Medical Intensive Care Unit, District Hospital Center, La Roche sur Yon (France), M. Fiancette MD Medical Intensive Care Unit, District Hospital Center, La Roche sur Yon (France), M. Henry Lagarrigue MD Medical Intensive Care Unit, District Hospital Center, La Roche sur Yon (France), J.C. Lacherade MD Medical Intensive Care Unit, District Hospital Center, La Roche sur Yon (France), 5 C. Lebert MD Medical Intensive Care Unit, District Hospital Center, La Roche sur Yon (France), L. Martin Lefevre MD Medical Intensive Care Unit, District Hospital Center, La Roche sur Yon (France), C. Pouplet MD Medical Intensive Care Unit, District Hospital Center, La Roche sur Yon (France), A. Seguin MD Medical Intensive Care Unit, District Hospital Center, La Roche sur Yon (France), I. Vinatier MD Medical Intensive Care Unit, District Hospital Center, La Roche sur Yon (France), A. Yehia MD Medical Intensive Care Unit, District Hospital Center, La Roche sur Yon (France), A. Bildea MD Medical Intensive Care Unit, General Hospital, Le Mans (France), S. Brodeur MD Medical Intensive Care Unit, General Hospital, Le Mans (France), B. Derrien MD Medical Intensive Care Unit, General Hospital, Le Mans (France), F. Grelon MD Medical Intensive Care Unit, General Hospital, Le Mans (France), M. Landais MD Medical Intensive Care Unit, General Hospital, Le Mans (France), C. Le Moal MD Medical Intensive Care Unit, General Hospital, Le Mans (France), P. Tirot MD Medical Intensive Care Unit, General Hospital, Le Mans (France), D. Vivier MD Medical Intensive Care Unit, General Hospital, Le Mans (France), G. Gasan MD Medical Intensive Care Unit, General Hospital, Lens (France), M. Jonard MD Medical Intensive Care Unit, General Hospital, Lens (France), M. Lemyze MD Medical Intensive Care Unit, General Hospital, Lens (France), J. Mallat MD Medical Intensive Care Unit, General Hospital, Lens (France), F. Pepy MD Medical Intensive Care Unit, General Hospital, Lens (France), J. Temime MD Medical Intensive Care Unit, General Hospital, Lens (France), D. Thevenin MD Medical Intensive Care Unit, General Hospital, Lens (France), P. Girardie MD Medical Intensive Care Unit, University Hospital, Lille (France), B. Voisin MD Medical Intensive Care Unit, University Hospital, Lille (France), E. Begot MD Medical Intensive Care Unit, University Hospital, Limoges (France), C. Chapellas MD Medical Intensive Care Unit, University Hospital, Limoges (France), M. Clavel MD Medical Intensive Care Unit, University Hospital, Limoges (France), T. Daix MD Medical Intensive Care Unit, University Hospital, Limoges (France), A. Fedou MD Medical Intensive Care Unit, University Hospital, Limoges (France), B. Francois MD Medical Intensive Care Unit, University Hospital, Limoges (France), A. Galy MD Medical Intensive Care Unit, University Hospital, Limoges (France), C. Gonzalez MD Medical Intensive Care Unit, University Hospital, Limoges (France), C. Hodler MD Medical Intensive Care Unit, University Hospital, Limoges (France), C. Mancia MD Medical Intensive Care Unit, University Hospital, Limoges (France), N. Pichon MD Medical Intensive Care Unit, University Hospital, Limoges (France), P. Vignon MD Medical Intensive Care Unit, University Hospital, Limoges (France), G. Belliard MD Medical Intensive Care Unit, General Hospital, Lorient (France), G. Grillet MD Medical Intensive Care Unit, General Hospital, Lorient (France), P. Bardou MD Medical Intensive Care Unit, General Hospital, Montauban (France), F. Bellec MD Medical Intensive Care Unit, General Hospital, Montauban (France), M. Bonnivard MD Medical Intensive Care Unit, General Hospital, Montauban (France), A. Marco MD Medical Intensive Care Unit, General Hospital, Montauban (France), J. Roustan MD Medical Intensive Care Unit, General Hospital, Montauban (France), S. Vimeux MD Medical Intensive Care Unit, General Hospital, Montauban (France), A. Delbove MDMedical Intensive Care Unit, University Hospital, Nantes (France), 6 N. Brule MD Medical Intensive Care Unit, University Hospital, Nantes (France), C. Bretonniere MD Medical Intensive Care Unit, University Hospital, Nantes (France), B. Gaborit MD Medical Intensive Care Unit, University Hospital, Nantes (France), C. Garret MD Medical Intensive Care Unit, University Hospital, Nantes (France), C. Guitton MD Medical Intensive Care Unit, University Hospital, Nantes (France), J.B. Lascarrou MD Medical Intensive Care Unit, University Hospital, Nantes (France), A. Le Meur MD Medical Intensive Care Unit, University Hospital, Nantes (France), J. Lorber MD Medical Intensive Care Unit, University Hospital, Nantes (France), D. Marest MD Medical Intensive Care Unit, University Hospital, Nantes (France), M. Martin MD Medical Intensive Care Unit, University Hospital, Nantes (France), L. Nicolet MD Medical Intensive Care Unit, University Hospital, Nantes (France), E. Pontis MD Medical Intensive Care Unit, University Hospital, Nantes (France), J. Reignier MD Medical Intensive Care Unit, University Hospital, Nantes (France), M. Vourc'h MD Medical Intensive Care Unit, University Hospital, Nantes (France), O. Zambon MD Medical Intensive Care Unit, University Hospital, Nantes (France), F. Barbier MD Medical Intensive Care Unit, Regional Hospital Center, Orléans (France), D. Benzekri MD Medical Intensive Care Unit, Regional Hospital Center, Orléans (France), T. Boulain MD Medical Intensive Care Unit, Regional Hospital Center, Orléans (France), A. Bretagnol MD Medical Intensive Care Unit, Regional Hospital Center, Orléans (France), A. Mathonnet MD Medical Intensive Care Unit, Regional Hospital Center, Orléans (France), G. Muller MD Medical Intensive Care Unit, Regional Hospital Center, Orléans (France), M. Nay MD Medical Intensive Care Unit, Regional Hospital Center, Orléans (France), I. Runge MD Medical Intensive Care Unit, Regional Hospital Center, Orléans (France), M. Skarzynski MD Medical Intensive Care Unit, Regional Hospital Center, Orléans (France), T. Kamel MD Medical Intensive Care Unit, Regional Hospital Center, Orléans (France), S. André MD Medical Intensive Care Unit, University Hospital, Paris (France), M. Arnaout MD Medical Intensive Care Unit, University Hospital, Paris (France), O. Ben Hadj Salem MD Medical Intensive Care Unit, University Hospital, Paris (France), S. Bourcier MD Medical Intensive Care Unit, University Hospital, Paris (France), A. Cariou MD Medical Intensive Care Unit, University Hospital, Paris (France), J.D. Chiche MD Medical Intensive Care Unit, University Hospital, Paris (France), J. Charpentier MD Medical Intensive Care Unit, University Hospital, Paris (France), M. Duprey MD Medical Intensive Care Unit, University Hospital, Paris (France), A. Ferre MD Medical Intensive Care Unit, University Hospital, Paris (France), L. Guillemet MD Medical Intensive Care Unit, University Hospital, Paris (France), M. Jamme MD Medical Intensive Care Unit, University Hospital, Paris (France), P. Jaquet MD Medical Intensive Care Unit, University Hospital, Paris (France), P. Jaubert MD Medical Intensive Care Unit, University Hospital, Paris (France), J. Llitjos MD Medical Intensive Care Unit, University Hospital, Paris (France), J.P. Mira MD Medical Intensive Care Unit, University Hospital, Paris (France), M. Paul MD Medical Intensive Care Unit, University Hospital, Paris (France), F. Pene MD Medical Intensive Care Unit, University Hospital, Paris (France), A. Roccabianca MD Medical Intensive Care Unit, University Hospital, Paris (France), G. Savary MD Medical Intensive Care Unit, University Hospital, Paris (France), S. Spagnolo MD Medical Intensive Care Unit, University Hospital, Paris (France), S. Valade MD Medical Intensive Care Unit, University Hospital, Paris (France), F. Ardisson MD Medical Intensive Care Unit, University Hospital, Pointe-à-Pitre (France), K. Elkoun MD Medical Intensive Care Unit, University Hospital, Pointe-à-Pitre (France), P. Lafforgue MD Medical Intensive Care Unit, University Hospital, Pointe-à-Pitre (France), B. Madeux MD Medical Intensive Care Unit, University Hospital, Pointe-à-Pitre (France), 7 P. Piednoir MD Medical Intensive Care Unit, University Hospital, Pointe-à-Pitre (France), B. Pons MD Medical Intensive Care Unit, University Hospital, Pointe-à-Pitre (France), G. Thiery MD Medical Intensive Care Unit, University Hospital, Pointe-à-Pitre (France), F. Boissier MD Medical Intensive Care Unit, University Hospital, Poitiers (France), S. Cabasson MD Medical Intensive Care Unit, University Hospital, Poitiers (France), D. Chatellier MD Medical Intensive Care Unit, University Hospital, Poitiers (France), R. Coudroy MD Medical Intensive Care Unit, University Hospital, Poitiers (France), J. Dufour MD Medical Intensive Care Unit, University Hospital, Poitiers (France), J. Frat MD Medical Intensive Care Unit, University Hospital, Poitiers (France), V. Goudet MD Medical Intensive Care Unit, University Hospital, Poitiers (France), M. Hoppe MD Medical Intensive Care Unit, University Hospital, Poitiers (France), A. Jamet MD Medical Intensive Care Unit, University Hospital, Poitiers (France), F. Joly MD Medical Intensive Care Unit, University Hospital, Poitiers (France), J. Marechal MD Medical Intensive Care Unit, University Hospital, Poitiers (France), R. Robert MD Medical Intensive Care Unit, University Hospital, Poitiers (France), A. Thille MD Medical Intensive Care Unit, University Hospital, Poitiers (France), A. Veinstein MD Medical Intensive Care Unit, University Hospital, Poitiers (France), P. Beuret MD Medical Intensive Care Unit, General Hospital, Roanne (France), J.C. Chakarian MD Medical Intensive Care Unit, General Hospital, Roanne (France), S. Devillez MD Medical Intensive Care Unit, General Hospital, Roanne (France), X. Fabre MD Medical Intensive Care Unit, General Hospital, Roanne (France), L. Gergelé MD Medical Intensive Care Unit, General Hospital, Roanne (France), B. Philippon-Jouve MD Medical Intensive Care Unit, General Hospital, Roanne (France), C. Brasse MD Medical Intensive Care Unit, General Hospital, Rodez (France), A. Delahaye MD Medical Intensive Care Unit, General Hospital, Rodez (France), J. Delmas MD Medical Intensive Care Unit, General Hospital, Rodez (France), S. Ena MD Medical Intensive Care Unit, General Hospital, Rodez (France), P. Letocart MD Medical Intensive Care Unit, General Hospital, Rodez (France), E. Moreau MD Medical Intensive Care Unit, General Hospital, Rodez (France), N. Barbarot MD Medical Intensive Care Unit, General Hospital, Saint Brieuc (France), J. Bousser MD Medical Intensive Care Unit, General Hospital, Saint Brieuc (France), A. Courte MD Medical Intensive Care Unit, General Hospital, Saint Brieuc (France), M. Debarre MD Medical Intensive Care Unit, General Hospital, Saint Brieuc (France), P. Fillatre MD Medical Intensive Care Unit, General Hospital, Saint Brieuc (France), A. Godard MD Medical Intensive Care Unit, General Hospital, Saint Brieuc (France), A. Goepp MD Medical Intensive Care Unit, General Hospital, Saint Brieuc (France), F. Legay MD Medical Intensive Care Unit, General Hospital, Saint Brieuc (France), J. Letheulle MD Medical Intensive Care Unit, General Hospital, Saint Brieuc (France), F. Marjot-zimbacca MD Medical Intensive Care Unit, General Hospital, Saint Brieuc (France), F. Mateos MD Medical Intensive Care Unit, General Hospital, Saint Brieuc (France), V. Botoc MD Medical Intensive Care Unit, General Hospital, Saint Malo (France), S. Chevalier MD Medical Intensive Care Unit, General Hospital, Saint Malo (France), P. Detouche MD Medical Intensive Care Unit, General Hospital, Saint Malo (France), J. Gouêllo MD Medical Intensive Care Unit, General Hospital, Saint Malo (France), N. Guinard MD Medical Intensive Care Unit, General Hospital, Saint Malo (France), C. Quentin MD Medical Intensive Care Unit, General Hospital, Saint Malo (France), P. Sichel MD Medical Intensive Care Unit, General Hospital, Saint Malo (France), N. Anca Raluca MD Medical Intensive Care Unit, University Hospital, Strasbourg (France), A. Berger MD Medical Intensive Care Unit, University Hospital, Strasbourg (France), 8 A. Boivin MD Medical Intensive Care Unit, University Hospital, Strasbourg (France), R. Clere-Jehl MD Medical Intensive Care Unit, University Hospital, Strasbourg (France), X. Delabranche MD Medical Intensive Care Unit, University Hospital, Strasbourg (France), S. Heenen MD Medical Intensive Care Unit, University Hospital, Strasbourg (France), J. Helms MD Medical Intensive Care Unit, University Hospital, Strasbourg (France), T. Khouri MD Medical Intensive Care Unit, University Hospital, Strasbourg (France), C. Kummerlen MD Medical Intensive Care Unit, University Hospital, Strasbourg (France), H. Merdji MD Medical Intensive Care Unit, University Hospital, Strasbourg (France), F. Meziani MD Medical Intensive Care Unit, University Hospital, Strasbourg (France), A. Monnier MD Medical Intensive Care Unit, University Hospital, Strasbourg (France), Y. Rabouël MD Medical Intensive Care Unit, University Hospital, Strasbourg (France), H. Rahmani MD Medical Intensive Care Unit, University Hospital, Strasbourg (France), D. Schnell MD Medical Intensive Care Unit, University Hospital, Strasbourg (France), L. Bodet Contentin MD Medical Intensive Care Unit, University Hospital, Tours (France), W. Darwiche MD Medical Intensive Care Unit, University Hospital, Tours (France), P. Dequin MD Medical Intensive Care Unit, University Hospital, Tours (France), S. Ehrmann MD Medical Intensive Care Unit, University Hospital, Tours (France), D. Garot MD Medical Intensive Care Unit, University Hospital, Tours (France), J. Grouille MD Medical Intensive Care Unit, University Hospital, Tours (France), A. Guillon MD Medical Intensive Care Unit, University Hospital, Tours (France), A. Joret MD Medical Intensive Care Unit, University Hospital, Tours (France), Y. Jouan MD MedicalIntensive Care Unit, University Hospital, Tours (France), A. Legras MD Medical 8Intensive Care Unit, University Hospital, Tours (France), C. Lhommet MD Medical Intensive Care Unit, University Hospital, Tours (France), J. Mankikian MD Medical Intensive Care Unit, University Hospital, Tours (France), E. Mercier MD Medical Intensive Care Unit, University Hospital, Tours (France), M. Morisseau MD Medical Intensive Care Unit, University Hospital, Tours (France), D. Perrotin MD Medical Intensive Care Unit, University Hospital, Tours (France), E . Rouve MD Medical Intensive Care Unit, University Hospital, Tours (France), C. Salmon-Gandonnière MD Medical Intensive Care Unit, University Hospital, Tours (France), F. Bruneel MD Medical Intensive Care Unit, General Hospital, Versailles (France), J. Fink MD Medical Intensive Care Unit, General Hospital, Versailles (France), A. Gros MD Medical Intensive Care Unit, General Hospital, Versailles (France), G. Lacave MD Medical Intensive Care Unit, General Hospital, Versailles (France), V. Laurent MD Medical Intensive Care Unit, General Hospital, Versailles (France), S. Legriel MD Medical Intensive Care Unit, General Hospital, Versailles (France), S. Merceron MD Medical Intensive Care Unit, General Hospital, Versailles (France), J. Moyer MD Medical Intensive Care Unit, General Hospital, Versailles (France), C. Simon MD Medical Intensive Care Unit, General Hospital, Versailles (France), G. Troché MD Medical Intensive Care Unit, General Hospital, Versailles (France), N. Zappella MD Medical Intensive Care Unit, General Hospital, Versailles (France), B. Zuber MD Medical Intensive Care Unit, General Hospital, Versailles (France).

## Credit Author Statement

Matthieu Petit and Guillaume Geri conceptualized the study.

Acquisition of data: Jean-Baptiste Lascarrou, Hamid Merdji, Gwenhael Colin and Alain Cariou acquired the data.

Matthieu Petit and Guillaume Geri did the formal analysis of the data.

Matthieu Petit and Guillaume Geri wrote the manuscript.

All authors reviewed the manuscript. ritical revision of the manuscript for important intellectual content: all authors.

Obtained funding: Jean-Baptiste Lascarrou obtained obtained funding for the original study.

Study was supervised by Guillaume Geri.
