# Supplementary material for: Hemodynamics and vasopressor support during targeted temperature management after cardiac arrest with non-shockable rhythm: A post hoc analysis of a randomized controlled trial
Source: Resusc Plus. 2022 Jul 12;11:100271. doi: 10.1016/j.resplu.2022.100271 (PMC9289859; doi:10.1016/j.resplu.2022.100271)
Supplement: Supplementary data 1 [file mmc1.pptx]

## Slide 1
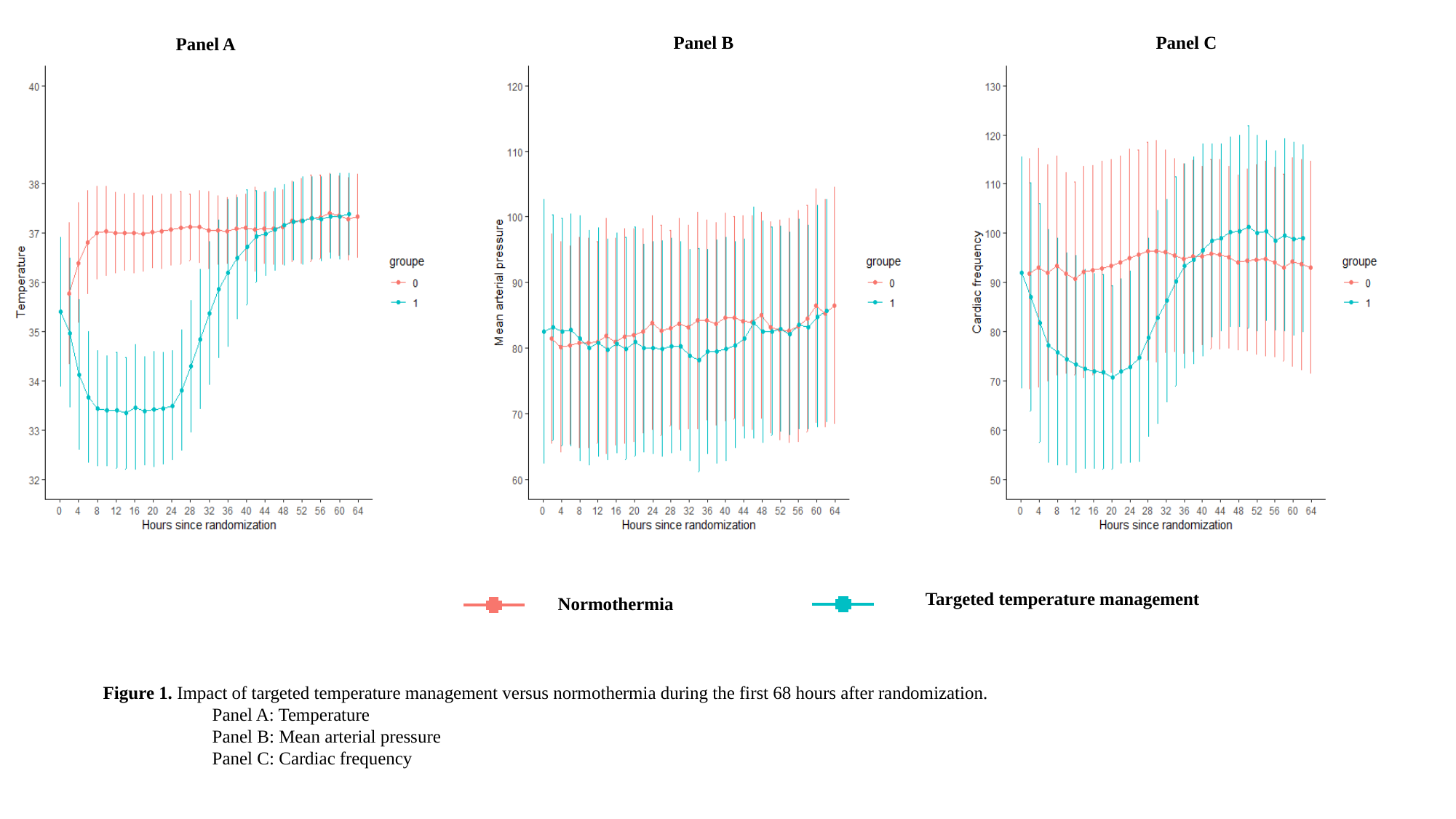

Panel B
Panel C
Panel A
Targeted temperature management
Normothermia
Figure 1. Impact of targeted temperature management versus normothermia during the first 68 hours after randomization.
	Panel A: Temperature
	Panel B: Mean arterial pressure
	Panel C: Cardiac frequency
