# Supplementary material for: Hemodynamics and vasopressor support during targeted temperature management after cardiac arrest with non-shockable rhythm: A post hoc analysis of a randomized controlled trial
Source: Resusc Plus. 2022 Jul 12;11:100271. doi: 10.1016/j.resplu.2022.100271 (PMC9289859; doi:10.1016/j.resplu.2022.100271)
Supplement: Supplementary data 2 [file mmc2.docx]

|  | **Targeted temperature management** | | **Normothermia** | |  |
| --- | --- | --- | --- | --- | --- |
|  | **No shock** | **Shock** | **No shock** | **Shock** |  |
| **n** | 101 | 183 | 94 | 203 |  |
| **Age - years** | 67.00 [57.00, 76.00] | 67.00 [58.00, 75.00] | 64.50 [57.00, 71.00] | 67.00 [58.00, 78.00] |  |
| **Sexe = male (%)** | 69 ( 68.3) | 116 ( 63.4) | 62 (66.0) | 126 (62.1) |  |
| **Charlson score** | 2.00 [0.00, 3.00] | 1.00 [0.00, 3.00] | 1.00 [0.00, 3.00] | 1.00 [0.00, 3.00] |  |
| **Chronic heart failure** | 14 ( 13.9) | 18 ( 9.8) | 6 ( 6.4) | 28 (13.8) |  |
| **Chronic respiratory disease** | 31 ( 47.0) | 66 ( 47.5) | 28 (41.8) | 79 (48.8) |  |
| **Location of cardiac arrest** |  |  |  |  |  |
| Home | 48 ( 47.5) | 90 ( 49.2) | 58 (61.7) | 99 (48.8) |  |
| Hospital | 26 ( 25.7) | 47 ( 25.7) | 19 (20.2) | 67 (33.0) |  |
| Public place | 27 ( 26.7) | 46 ( 25.1) | 17 (18.1) | 37 (18.2) |  |
| **Bystander-witnessed cardiac arrest** | 96 ( 95.0) | 178 ( 97.3) | 86 (91.5) | 187 (92.6) |  |
| **Bystander-performed CPR** | 68 ( 70.8) | 132 ( 74.6) | 60 (69.8) | 147 (78.2) |  |
| **Cause of cardiac arrest** |  |  |  |  |  |
| Asphyxia | 55 ( 54.5) | 90 ( 49.2) | 14 (15.1) | 22 (12.0) |  |
| Other medical cause | 8 ( 7.9) | 30 ( 16.4) | 48 (51.1) | 97 (47.8) |  |
| Cardiac cause | 27 ( 26.7) | 49 ( 26.8) | 15 (16.0) | 29 (14.3) |  |
| Pulmonary embolism | 4 ( 4.0) | 6 ( 3.3) | 22 (23.4) | 55 (27.1) |  |
| Neurological cause | 3 ( 3.0) | 4 ( 2.2) | 0 ( 0.0) | 11 ( 5.4) |  |
| Drowning | 1 ( 1.0) | 3 ( 1.6) | 2 ( 2.1) | 4 ( 2.0) |  |
| Hanging | 3 ( 3.0) | 0 ( 0.0) | 0 ( 0.0) | 2 ( 1.0) |  |
| Trauma | 0 ( 0.0) | 1 ( 0.5) | 6 ( 6.4) | 4 ( 2.0) |  |
| **No_flow - min** | 2.50 [0.00, 6.25] | 2.00 [0.00, 5.00] | 5.00 [0.00, 7.00] | 0.00 [0.00, 5.00] |  |
| **Low_flow - min** | 15.50 [10.00, 20.00] | 15.00 [10.00, 26.00] | 17.50 [10.00, 25.00] | 18.00 [10.00, 26.50] |  |
| **Use of epinephrin** | 89 ( 88.1) | 170 ( 92.9) | 84 (89.4) | 192 (94.6) |  |
| **Coronary angiogrpahy** | 26 ( 25.7) | 49 ( 26.8) | 20 (21.3) | 53 (26.1) |  |
| **Temperature at enrollment - °C** | 35.50 [34.50, 36.40] | 35.60 [34.80, 36.50] | 35.45 [34.52, 36.50] | 35.40 [34.32, 36.50] |  |
| **Cardiovascular SOFA at enrollment** |  |  |  |  |  |
| 0 | 35.70 [35.00, 36.40] | 35.50 [34.23, 36.40] | 63 (67.0) | 0 ( 0.0) |  |
| 1 |  |  | 31 (33.0) | 0 ( 0.0) |  |
| 2 | 68 ( 67.3) | 0 ( 0.0) | 0 ( 0.0) | 0 ( 0.0) |  |
| 3 | 30 ( 29.7) | 0 ( 0.0) | 0 ( 0.0) | 27 (13.3) |  |
| 4 | 3 ( 3.0) | 0 ( 0.0) | 0 ( 0.0) | 176 (86.7) |  |
| **Brain death** | 14 (16.5) | 32 (21.6) | 19 (24.1) | 28 (16.5) |  |
| **CPC score of 1 or 2 on day 90** | 8 ( 7.9) | 21 (11.5) | 4 ( 4.3) | 13 ( 6.4) |  |
| **CPC score distribution on day 90** |  |  |  |  |  |
| **1** | 4 ( 4.0) | 12 ( 6.6) | 3 ( 3.2) | 8 ( 3.9) |  |
| **2** | 4 ( 4.0) | 9 ( 4.9) | 1 ( 1.1) | 5 ( 2.5) |  |
| **3** | 9 ( 8.9) | 13 ( 7.1) | 11 (11.7) | 20 ( 9.9) |  |
| **4** | 0 ( 0.0) | 1 ( 0.5) | 0 ( 0.0) | 0 ( 0.0) |  |
| **5** | 84 (83.2) | 148 (80.9) | 79 (84.0) | 170 (83.7) |  |
| **Death by day 90** | 83 (82.2) | 148 (80.9) | 77 (81.9) | 170 (83.7) |  |

**ESM Table 1. Baseline characteristics and outcome of patients according to shock status at admission and intervention.**

*Categorical variables are expressed as number (%) and continous variables as median (interquartile ranges).*

*SOFA, Sequential organ failure assessment, Cerebral performance category*

|  | **Circulatory failure after intervention** |
| --- | --- |
| **n** | 20 |
| **Age - years** | 55.00 [50.50, 62.50] |
| **Sexe = male (%)** | 15 ( 78.9) |
| **Charlson score** | 0.00 [0.00, 1.00] |
| **Chronic heart failure** | 0 ( 0.0) |
| **Chronic respiratory disease** | 7 ( 53.8) |
| **Location of cardiac arrest** |  |
| Home | 11 ( 57.9) |
| Hospital | 3 ( 15.8) |
| Public place | 5 ( 26.3) |
| **Bystander-witnessed cardiac arrest** | 17 ( 89.5) |
| **Bystander-performed CPR** | 12 ( 70.6) |
| **Cause of cardiac arrest** |  |
| Asphyxia | 9 ( 47.4) |
| Other medical cause | 4 ( 21.1) |
| Cardiac cause | 2 ( 10.5) |
| Pulmonary embolism | 0 ( 0.0) |
| Neurological cause | 1 ( 5.3) |
| Drowning | 1 ( 5.3) |
| Hanging | 2 ( 10.5) |
| Trauma | 0 ( 0.0) |
| **No_flow - min** | 2.00 [0.00, 5.00] |
| **Low_flow - min** | 20.00 [14.50, 30.00] |
| **Use of epinephrin** | 15 ( 78.9) |
| **Coronary angiogrpahy** | 5 ( 26.3) |
| **Temperature at enrollment - °C** | 35.20 [34.70, 35.65] |
| **Targeted temperature management** | 9 ( 52.9) |
| **Brain death** | 9 ( 52.9) |
| **CPC score of 1 or 2 on day 90** | 2 ( 10.5) |
| **CPC score distribution on day 90** |  |
| **1** | 2 ( 10.5) |
| **2** | 0 ( 0.0) |
| **3** | 0 ( 0.0) |
| **4** | 0 ( 0.0) |
| **5** | 17 ( 89.5) |
| **Death by day 90** | 9 ( 52.9) |
|  |  |

**ESM Table 2. Characteristics and outcome of patient who developped circulatory failure after intervention.**

*Categorical variables are expressed as number (%) and continous variables as median (interquartile ranges).*

*SOFA, Sequential organ failure assessment, CPC Cerebral performance category*
